# Balance and Vestibular Deficits in Pediatric Patients with Autism Spectrum Disorder: An Underappreciated Clinical Aspect

**DOI:** 10.1155/2022/7568572

**Published:** 2022-08-16

**Authors:** Linda M. Oster, Guangwei Zhou

**Affiliations:** ^1^Department of Otolaryngology and Communication Enhancement, Boston Children's Hospital, Boston, MA, USA; ^2^Department of Otolaryngology, Harvard Medical School, Boston, MA, USA

## Abstract

Children with autism spectrum disorder (ASD) not only have communication and social difficulties, but also exhibit poor balance and motor control ability, which frequently affect daily activities. Effective balance and motor control rely on the integration of somatosensory, visual, and vestibular inputs. Although reports of balance dysfunction in ASD have been documented, comprehensive studies of balance and vestibular function in children with ASD are scarce. In this study, we retrospectively reviewed 36 pediatric patients diagnosed with ASD who underwent balance/vestibular laboratory testing in our speciality clinic. Results from sensory organization test (SOT) or modified clinical test for sensory integration of balance (mCTSIB) found that out of 15 patients, 80% had abnormal findings. Of the children who successfully completed each vestibular test, abnormal responses were observed in 12 (80%) sensory organization tests, 5 (24%) vestibular evoked myogenic potential (VEMP), 22 (66%) videonystagmography (VNG), and 11 (32%) sinusoidal rotary chair tests. These results indicate that balance and vestibular testing may be of diagnostic value for clinicians and providers as an aid in early detection, intervention, and the development of appropriate management and therapies for this patient population. Increased awareness of this topic is warranted to promote better clinical management of this special group of patients and improve their quality of life.

## 1. Introduction

The primary features of autism spectrum disorder (ASD) include impaired communication, decreased social reciprocity, and restricted behavior [1]. While most are familiar with these aspects, other sensory deficits or impairments may also manifest in children with ASD. In recent years, there has been increasing evidence that the ASD not only affects communication, cognition, mood/emotion, and behavior but also affects motor control and balance function. For instance, it was reported that motor deficits were so prevalent in the ASD that they could be considered a “potential core feature of the ASD” [2]. In fact, it has been suggested that patients with ASD have a “whole range of subtle motor control deficits (including postural instability)” which impacts their cognitive and social development [3]. Furthermore, it has been described that ASD can be characterized by a range of motor control deficits [4] and sensory subtypes [5].

While posture/balance abnormalities have been reported to be among the symptoms of ASD [6], many have primarily attributed this balance problem to poor motor control/skills. Therefore, many balance assessment tools have since focused on measures of gross motor skill proficiency. However, this type of approach neglects the role of vestibular contribution to balance function. To maintain postural stability and normal balance, visual, somatosensory, and vestibular inputs must be accurately acquired and processed in real time. The vestibular system plays a key role especially when visual input and/or somatosensory information is lacking or unreliable. However, clinical studies of vestibular function in ASD are limited. Previously, vestibular testing including random saccades, smooth pursuit, and rotary chair was conducted, and delayed latency in saccades was reported [7]. In contrast, no difference in vestibulo-ocular reflex (VOR) between children with ASD and typical development controls was found. A later study looking at the VOR in children with ASD found abnormalities, which were attributed to alternations in cerebellar and brainstem circuitry [8]. Central vestibular dysfunction in ASD was highlighted in a recent review study published in 2021 [9].

Considering the limited and conflicting nature of current literature regarding balance and vestibular function in ASD, a more thorough and detailed investigation is desirable. In addition, cohorts in previous studies were not restricted to pediatric patients with ASD. To have a better understanding of balance and vestibular function in children with ASD, we conducted a retrospective review of balance and vestibular testing outcomes. We hope to increase awareness of the presence of balance and vestibular impairment in this particular group of children, which may promote better clinical care and improve their quality of life.

## 2. Methods

### 2.1. Participants

A total of 36 pediatric patients (17 males and 19 females) were included in this study. All participants included had documented clinical diagnoses of ASD, commonly given by Neurology and/or Developmental Medicine prior to referral for vestibular/balance testing. Patients' age ranged from 2 to 18 years, with an average of 8.4 (SD = 8.5) years. Patients were grouped by age in the following ways based on the date they were tested, to facilitate viewing demographic data: Group 1 (2–6 years), Group 2 (7–12 years), and Group 3 (13–18 years). Out of the 36 total patients, 9 (3 in each age group) were identified with hearing loss. In contrast, about 25 patients (about 70%) had documented motor delays. More children with motor delays were found in Group 1 compared to Groups 2 and 3. Similarly, 30 patients (about 83%) had delayed speech/language or were nonverbal, and the majority of these patients were found in Group 1. These findings are summarized in Table 1. All patients that underwent testing were able to understand and follow simple directions as given by the clinicians.

Reasons for balance and vestibular testing can be classified as the following: balance complaints, such as balance concerns or frequent falls, and vestibular complaints, such as dizziness, vertigo, or motion sickness. There were 27 patients who came in for evaluation because of balance complaints alone and 3 with only vestibular complaints. Five patients had both balance and vestibular complaints. In addition, two patients were also noted for regression in activity. This retrospective study was approved by the institutional review board of Boston Children's Hospital.

### 2.2. Testing Procedures

All patients underwent age-appropriate balance and vestibular testing to the best of their ability in our vestibular testing laboratory. Balance testing includes a sensory organization test (SOT) or a modified clinical test for sensory integration of balance (mCTSIB) using the NeuroCom Smart EquiTest system, depending upon their age and ability to follow instructions. Vestibular testing included videonystagmography (VNG), sinusoidal rotary chair test (using Micromedical System 2000), vestibular evoked myogenic potentials (VEMP) using the Biologic NavigatorPro evoked potential recording system, and video-head impulse testing (vHIT) using the ICS Impulse system. VEMP testing was performed by a licensed audiologist and a trained assistant according to the procedure described in detail in our previous work [10]. All other vestibular testing was performed according to protocols also detailed previously [11, 12]. All systems used were from Natus Medical Inc., except for VNG and rotary chair systems.

Extensive pediatric testing often requires extra measures to ensure testing is a successful experience [13]. Increased communication with both parent and child about what each test involves beforehand is essential. In vestibular/balance testing, this often involves the clinician acting out instructions or demonstrating activities in a child-friendly manner. In addition, the clinicians will engage in play with the patient (pretend play, engaging in conversation, reading a story, and playing with toys) to allow time for the child to become comfortable with both the testing environment and the testing staff. During testing, parental involvement is crucial to allow for soothing, encouragement, and positive reinforcement. Children are given ample breaks both during testing and between tests, and are reminded they can let testing staff or their parents know if they are tired and need a break. Rewards such as snacks given by parents, happy music during sinusoidal rotary chair testing, and stickers given by the clinician can help all children successfully complete extensive testing. Most importantly, practicing patience is critical for a successful vestibular/balance assessment. Due to patient refusal, fatigue, fear, or inattention, not every patient completed each test in this study, which can be commonplace in a pediatric speciality clinic. Lastly, newer vestibular tests such as vHIT, for example, were not available in our clinic until recently; therefore, only 8 patients underwent this test.

### 2.3. Data Analysis

Balance and vestibular testing outcomes were classified as abnormal if results lied outside the norms provided by the manufacturers, except for VEMP, in which the outcome was defined by our institutional age-specific norms [10]. The percentage of abnormal findings in each test was highlighted. Due to a relatively small sample size, no additional statistical analysis was conducted.

## 3. Results

### 3.1. Balance Testing

A composite score was generated by NeuroCom software after all six SOT conditions were completed. This score is marked as normal/abnormal by the software based on age-specific norms, and we used this score to define the test outcome as either normal or abnormal. For patients under the age of 3 or who were unable to finish SOT, mCTSIB was used as an alternative balance test. Overall, 15 patients were able to successfully complete balance testing and 12 (80%) had abnormal outcomes.

### 3.2. Vestibular Testing

In VNG testing, random saccades, smooth pursuit, and optokinetic data were collected and automatically analyzed by Micromedical software. Out of 33 patients who were able to complete VNG testing, 22 (66%) had abnormal findings. For the sinusoidal rotary chair test, VOR gain, phase, and fixation suppression data were collected and used as criteria for abnormality. A total of 34 patients completed sinusoidal rotary chair testing, and 11 (32%) had abnormal outcomes. In VEMP testing, the threshold and amplitude of the responses were measured and the outcome was classified as abnormal/normal based on institutional norms. Among the 21 children who completed VEMP testing, only 5 (24%) were abnormal. As a new tool in vestibular testing, the abnormalities identified by vHIT are defined as reduced VOR gain and/or the presence of corrective saccades. We found that 3 patients out of 8 who completed the test (about 38%) had abnormal results.

**Figure 1:**
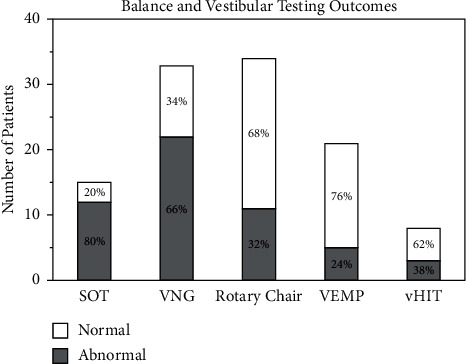
Bar chart depicting the testing outcome, reported as the percent of patients scoring abnormally (dots) or normally (stripes) for each test. From left to right, the percentage of patients that scored abnormally in each test is as follows: 80%, 66%, 32%, 24%, and 38%.

A summary of balance and vestibular testing outcomes can be found in Figure 1. Overall, 14 out of the total 36 patients, i.e., about 39%, had multiple abnormal findings in balance and vestibular testing.

## 4. Discussion

Our retrospective review aimed to raise awareness of balance deficits among children with ASD. We also try to address all possible factors that may affect balance, especially vestibular functional status. First of all, a majority of our children with ASD were found to have balance impairments, demonstrated by an abnormal SOT and/or mCTSIB. These two tests assess patients' ability to integrate visual, vestibular, and somatosensory inputs to maintain balance. Since children with ASD are known to have abnormalities in sensory integration [4, 6], these findings are not surprising. The effect of ASD on sensory integration in balance has been emphasized previously in the literature [14]. We also noticed oculomotor dysfunction (demonstrated in random saccades, smooth pursuit, and optokinetic testing) in children with ASD. For example, increased latency in saccades, or failure to track the target during pursuit test consistently and/or smoothly was noticed by the clinicians. Reduced gain of optokinetics was also noticed. All of these findings indicate central involvement, consistent with a previous report [15].

The VOR is responsible for stabilizing images on the retina during head movements. The sinusoidal rotary chair test, the standard procedure for VOR testing, can assess not only the functional status of the peripheral vestibular system (e.g., the lateral semicircular canal) but also sensory integration in the central vestibular pathway. The conflicting findings reported by several publications in the past motivated us to search for more conclusive VOR results from our pediatric patients [7, 8, 16, 17]. Interestingly, we found a minority of patients, i.e., less than a third, had abnormal VOR measurements (gain, phase, etc.). This finding may implicate a diversity of underlying pathophysiology in this special group of patients and thus highlights the importance of evaluating the VOR status in children with ASD. Proper intervention, based on the outcomes of VOR, should be provided to address patients' individual needs rather than using a fixed approach.

The usefulness of vHIT for ASD was reported by a recent vHIT study conducted in pediatric patients with neurodevelopmental disorders (NDDs) including ASD, compared to a group of children with typical development [18]. A significant difference in vHIT outcome was observed among the two groups and this was characterized as “a potential biomarker” for underlying NDDs. As a newer VOR testing procedure, the vHIT can assess all six semicircular canals individually and is well-tolerated by pediatric patients [19]. In our study, 8 patients completed vHIT and 3 had abnormal results, i.e., reduced VOR gain and/or the presence of corrective saccades. Due to the relatively small number of patients involved in this study, the true usefulness of vHIT may not be exemplified here, and more vHIT testing in children with ASD is needed for a conclusive answer. Clinically, the VEMP test is used to evaluate the function of the otolith organs and vestibular nerves. Cervical VEMP, which is complementary to the sinusoidal rotary chair and vHIT testing, can assess saccular function specifically, allowing for a more comprehensive vestibular evaluation [10]. Although there is a lack of VEMP research in children with ASD in the literature, we found that about a quarter of our patients indeed had cervical VEMP abnormalities. At this time, we are unable to provide a definitive explanation for such a finding; therefore, future study on this topic is justified.

Although children with ASD may be frequent hospital visitors for a variety of reasons such as neurological and developmental care, they rarely come in for a balance/vestibular evaluation exclusively. One of the main reasons is that these patients often are unable to describe their own symptoms and/or communicate with caregivers effectively and readily even while experiencing vestibular disturbances. As demonstrated in our results, many ASD patients, especially the younger ones, have speech/language delays or are nonverbal. In this situation, parents and caregivers need to pay attention to any possible symptoms and advocate for them so that a referral for balance and vestibular testing can be made. Signs or symptoms include (but are not limited to) delayed motor milestones, late walking, frequent tripping or falling, poor coordination, and/or clumsiness that affects daily activities. Therefore, increased awareness of balance and vestibular problems in children is needed. As stated in a recent publication [9], “vestibular issues are likely under-reported in children with ASD and may go unrecognized”. That being said, balance and vestibular testing can be very challenging for any child with ASD. These patients often have sensory issues and anxiety when participating laboratory testing. Proper procedural modifications and use of a child-friendly approach during evaluation are imperative. For example, giving patient sufficient break between testing and encouragement to participate the testing may be necessary. Occasionally, conducting tests as playing game with children can be helpful as well. With a comprehensive evaluation, early detection of balance and vestibular deficits in children with ASD makes necessary interventions such balance training, vestibular rehab, and personalized evidence-based physical therapy interventions possible, which will lead to improvement of their quality of life [20, 21]. Lastly, it is worth noting that our study includes the fact that this group of patients with ASD have a higher occurrence of vestibular/balance impairment than the general population, probably due to the fact that they were referred for testing because of signs or complaints of vestibular/balance impairment. Future studies may replicate the methods discussed previously in a randomized population of children with ASD to determine the fraction of children with ASD who have balance/vestibular impairments.

In conclusion, the results of this retrospective review indicate that balance impairment and vestibular deficits are present in children with ASD, and this may contribute to a whole host of symptoms and challenges faced each day from a very early age such as activity avoidance (decreased confidence and fear), social isolation, and increased risk of physical injury such as falling. We not only hope to underline that clinical evaluation of balance and vestibular function in ASD patients is diagnostically valuable, but aims to raise awareness of this important issue so that clinical care providers for children with ASD can identify signs or symptoms of possible balance/vestibular impairment.

## Figures and Tables

**Table 1:** Background characteristics by age group.

Age (yrs)	Sex		Hearing loss		Motor delay		Speech/Language delay		
	Female	Male	Yes	No	Yes	No	Normal	Delayed	Nonverbal
2–6	8	8	3	13	11	5	0	15	1
7–12	8	4	3	9	7	5	3	8	1
13–18	3	5	3	5	7	1	3	3	2
Total	19	17	9	27	25	11	6	26	4

## Data Availability

The data used to support the findings of this study are available on request from the corresponding author. The data are not publicly available due to privacy or ethical restrictions.

## Additional Points

*Lay Summary*. Children diagnosed with autism spectrum disorder (ASD) are often faced with balance and motor control impairment. Our retrospective review of balance and vestibular assessment of children with ASD showed that the majority of ASD patients referred for testing scored abnormally on balance testing. Increased awareness of this topic will promote better clinical management and improve their quality of life.
